# DNA demethylating agent decitabine broadens the peripheral T cell receptor repertoire

**DOI:** 10.18632/oncotarget.9352

**Published:** 2016-05-13

**Authors:** Jing Nie, Yan Zhang, Xiang Li, Meixia Chen, Chuanjie Liu, Weidong Han

**Affiliations:** ^1^ Department of Immunology, Institute of Basic Medical Science, PLA General Hospital, Beijing, 100853, China; ^2^ Department of Biological Therapy, PLA General Hospital, Beijing, 100853, China

**Keywords:** T cell receptor repertoire, pharmacometabonomics, decitabine, epigenetic therapy, solid tumor

## Abstract

**Purpose:**

Decitabine, a promising epi-immunotherapeutic agent has shown clinical responses in solid tumor patients, while the anti-tumor mechanisms were unclear. We aimed to investigate the immunomodulatory effect of decitabine in peripheral T cells.

**Experimental design:**

We applied next-generation sequencing to investigate the complementarity-determining region 3 (CDR3) of the TCRβ gene, the diversity of which acts as the prerequisite for the host immune system to recognize the universal foreign antigens. We collected the peripheral blood mononuclear cells (PBMCs) from 4 patients, at baseline and after 2 cycles of low-dose decitabine therapy.

**Results:**

An increase of the unique productive sequences of the CDR3 of TCRβ was observed in all of the 4 patients after decitabine treatment, which was characterized by a lower abundance of expanded clones and increased TCR diversity compared with before decitabine treatment. Further analysis showed a tendency for CD4 T cells with an increased CD4/CD8 ratio in response to decitabine therapy. In addition, the genome-wide expression alterations confirmed the effects of decitabine on immune reconstitution, and the increase of TCR excision circles (TRECs) was validated.

**Conclusions:**

The low-dose DNMT inhibitor decitabine broadens the peripheral T cell repertoire, providing a novel role for the epigenetic modifying agent in anti-tumor immune enhancement.

## INTRODUCTION

B and T lymphocytes possess the capacity to recognize foreign molecules through various antigen-binding receptors (B cell receptor and T cell receptor [TCR]). The diversity of immune repertoires is created via recombination of variable (V), diversity (D), and joining (J) gene segments, which form the antigen-binding variable region. Moreover, nucleotide deletion or insertion at gene junction sites contributes to the substantial TCR diversity. As many as 10^11^ different TCRs can be generated during the somatic recombination process in human T cells [1]. The antigenic specificity of T lymphocytes is primarily determined by the sequences in the hypervariable CDR3 (complementarity-determining region 3) of the TCR. The CDR3 sequences have been viewed as the natural identifier of T cell clonality.

In recent years, the application of deep sequencing technology has allowed for the quantitative analysis of the frequencies of distinct TCR clonotypes, providing precise measurement of TCR diversity [2, 3]. The peripheral blood includes a substantial repertoire of T lymphocytes that gain the ability to recognize various antigens. The constriction of the TCR repertoire could cause impaired immune response to viral and bacterial infections [4], poor reaction to vaccination [5], and delayed immune recovery after chemotherapy and hematopoietic stem cell transplantation [6, 7]. Especially, reduced TCR diversity was observed in multiple tumor patients [8, 9]. In addition, the roughly linear decrease of TCR diversity with aging was confirmed using high-throughput sequencing technology, and the TCR repertoire diversity was correlated with the percentage of naïve T cells [10].

Epigenetic modifying agents have been approved in the treatment of hematological diseases, and various recent studies reported the clinical benefits of using low doses of DNA demethylating agent decitabine in solid tumor patients [11–14]. It has been widely reported that the epigenetic modifying agents enhanced the sensitivity of tumor cells to chemotherapeutic drugs, and that lower-dose decitabine treatment alone or combined with adoptive immunotherapy resulted in beneficial clinical responses in patients with advanced hepatocellular carcinoma (HCC) [15, 16]. Decitabine could enhance tumor antigen expression and endogenous antigen processing, increase the expression of MHC and co-stimulatory molecules, and boost effector T cell function [12, 17]. Recently, it was reported that DNA demethylating drugs can augment anti-tumor immune response via activating the expression of endogenous retroviruses by targeting cancer cells [18]. However, the roles and mechanism of low-dose decitabine in solid tumor treatment, especially for the host T cells, were unclear. In this study, we aimed to explore the effect of low-dose decitabine on T-cell immune repertoire.

## RESULTS

### Global turnover of the T cell repertoire

To identify the effects of low-dose decitabine (DAC) treatment on T cell repertoire, we performed deep sequencing of the TCRβ CDR3 repertoire in PBMCs from 4 solid tumor patients before and after 2 cycles of low-dose decitabine treatment, including 2 patients with hepatocarcinoma, 1 with ovarian cancer, and another with carcinoma of the uterine tube (Accession number SRP071826). Through sequencing and analyzing, a total of about 14 million good sequencing read distributed among approximately 45,000 TCRβ rearrangement clones, which is summarized in Supplementary Table 1. Among the total TCR sequences, all 50 Vβ genes, 2 Dβ genes, and 13 Jβ genes were detected, suggesting that our sequencing method could cover the entire TCRβ repertoire. The usage frequency of the various TCRβV and TCRβJ genes in each patient are listed in Supplementary Table 2.

**Figure 1:**
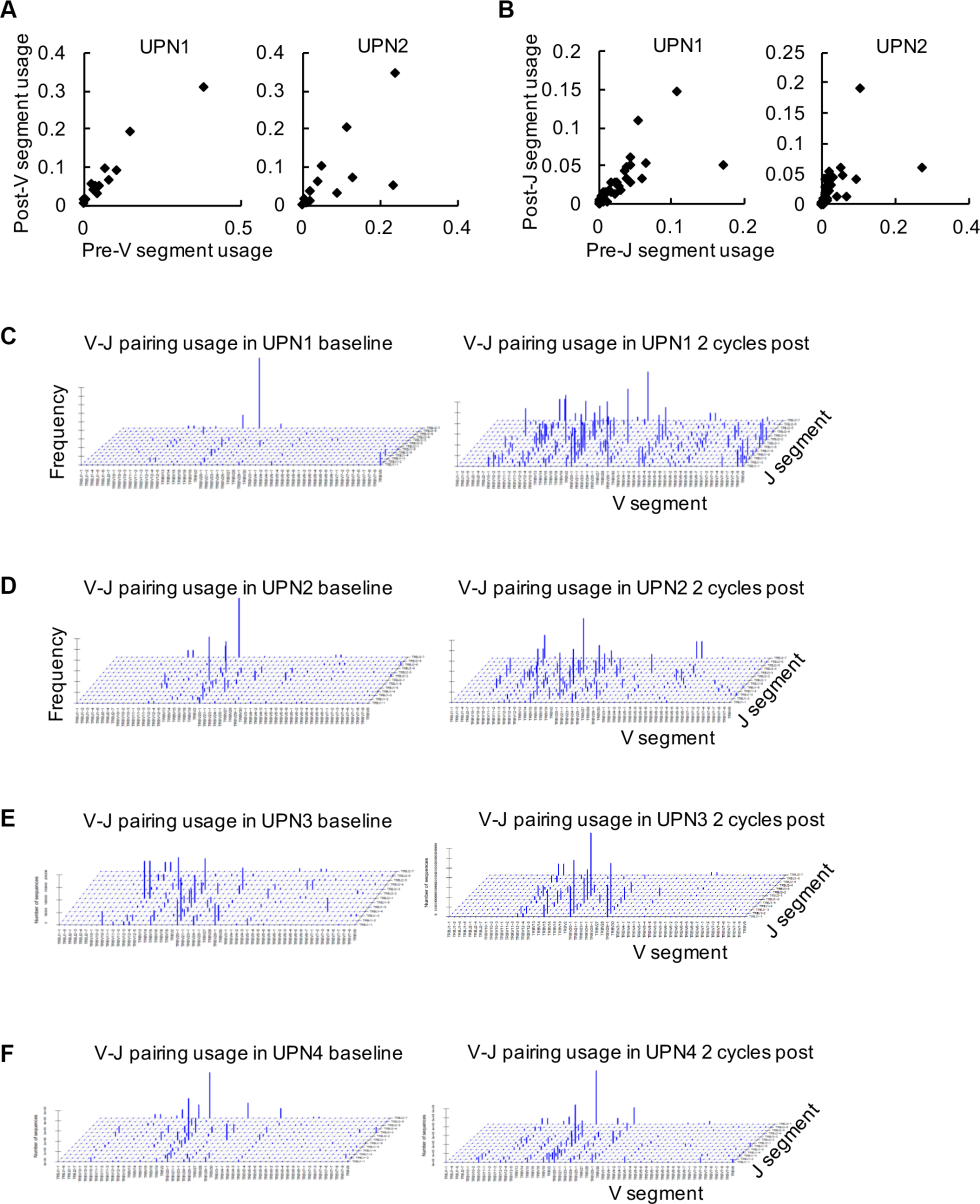
Global analysis of V_β_-J_β_ gene segment combinations (**A**, **B**) The frequencies of TCRβ V (A) and J (B) gene segments in peripheral blood samples at baseline (pre) and following 2 cycles of decitabine therapy (post) from UPN1 and UPN2 are shown. (**C**–**F**) Frequencies of specific V_β_-J_β_ gene segment combinations in TCRβ CDR3 sequences are expressed in UPN1 (C), UPN2 (D), UPN3 (E), and UPN4 (F) before and after decitabine therapy.

According to our data, we observed that the usage frequencies of V and J genes were changed in blood samples before and after decitabine therapy, including both expansion and loss of T cell clonotypes (Figure 1A and 1B). To visualize the V-J usage pattern (for the different combinations of all 50 V genes with any one of the 13 J genes), 3-D reconstruction was performed. As shown in Figure 1C to 1F, the relative dominance of limited V-J combinations was observed in the TCR repertoire of these 4 patients at baseline, while a global turnover with more balanced V-J usage was observed following 2 cycles of low-dose decitabine therapy.

**Figure 2:**
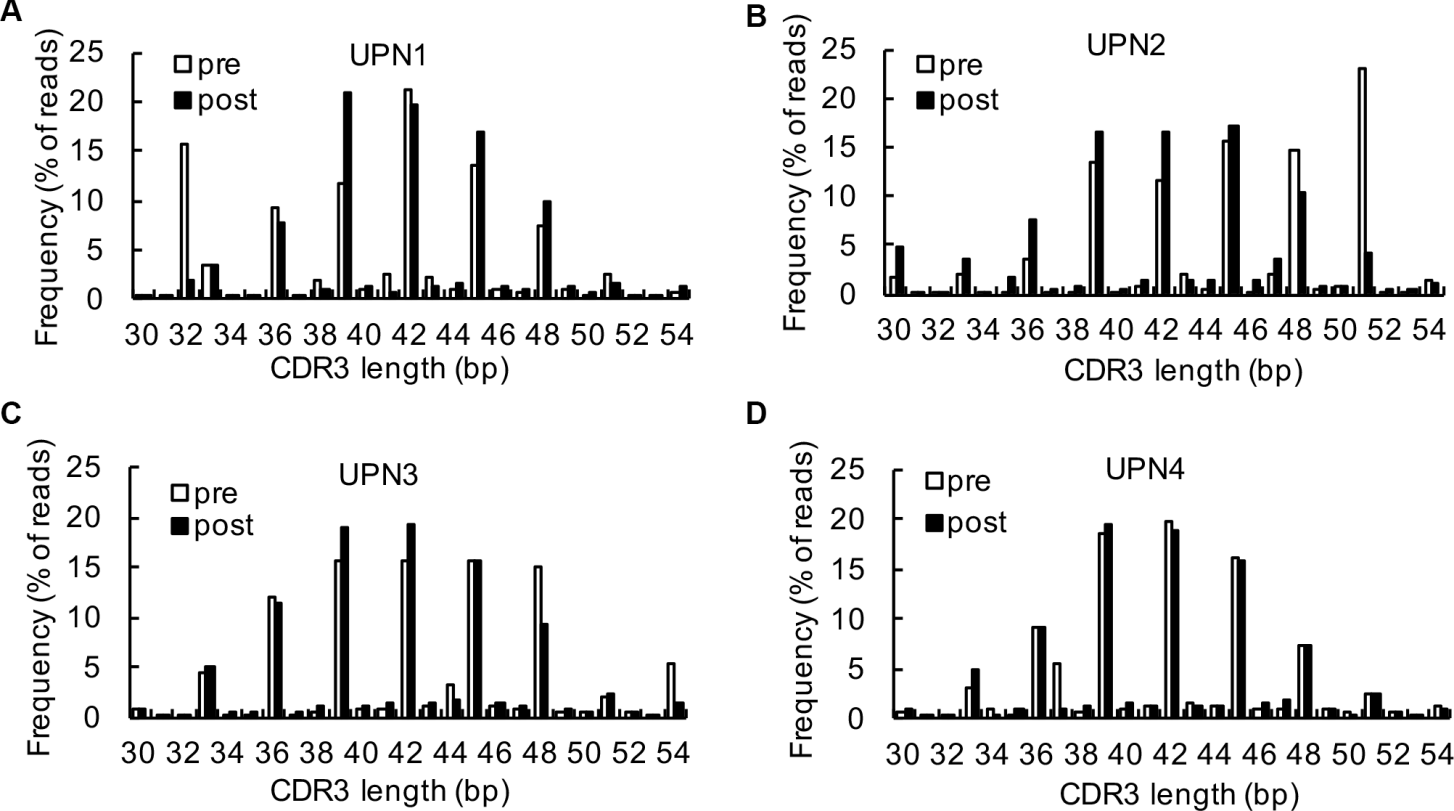
Analysis of CDR3 spectrums (**A**–**D**) A comparison of CDR3 region length and distribution between baseline and after 2 cycles of decitabine therapy in UPN1 (A), UPN2 (B), UPN3 (C), and UPN4 (D).

Clonal T cell expansion determines CDR3 size distribution patterns. In blood from healthy donors, most profiles reflecting the CDR3 length diversity showed 5 to 8 peaks at 3 nucleotide intervals, with a nearly Gaussian distribution. Nevertheless, only one or a few dominant peaks indicated that there were one or several cDNA with the same size or similar junctional regions, which referred to clonal T-cell expansion [19]. In our study, the CDR3 size spectratype profiles showed that most of the CDR3 lengths spanned from 33 to 51. The previous CDR3 length distribution pattern at baseline was an abnormal distribution; however, the CDR3 length distribution was more symmetric with the highest fraction at 39 following decitabine therapy (Figure 2A to 2D). These results indicated a significant global turnover of the T cell repertoire.

### Changes in relative TCRβ V and TCRβ J usage frequency

**Figure 3:**
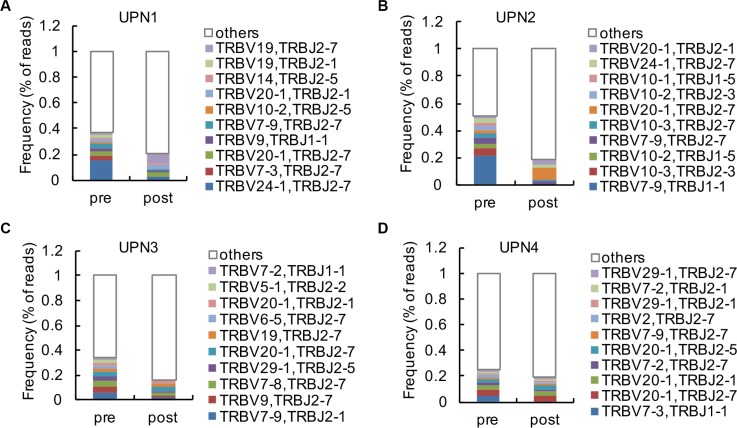
Analysis of high clonal V-J combinations (**A**–**D**) The 10 most frequent V-J combinations of PBMCs from UPN1 (A), UPN2 (B), UPN3 (C) and UPN4 (D) at baseline and after 2 cycles of decitabine therapy are indicated in color; the remaining V-J combinations are grouped in white.

**Figure 4:**
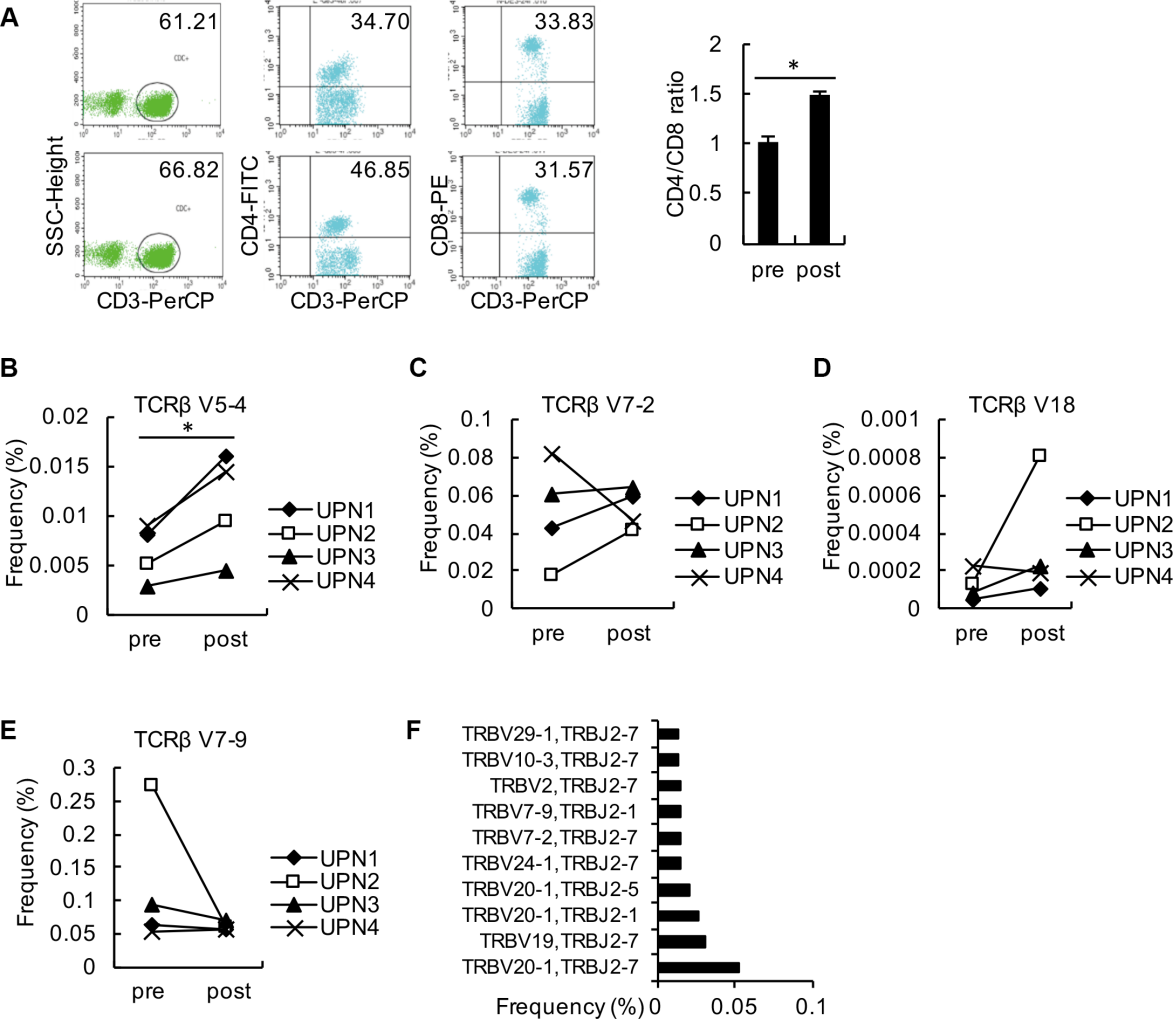
Analysis of changes in relative TCRβ V and TCRβ J (**A**) PBMCs from the 4 patients were collected as detected by flow cytometry using antibodies against CD3, CD4, and CD8. The average ratios of CD3+CD4+: CD3+CD8+ from these patients are shown. (**B**–**E**) The frequencies of TCRβ V5-4 (B), TCRβ V7-2 (C), TCRβ V18 (D) and TCRβ V7-9 (E) in PBMCs from each patient before and after decitabine therapy are shown. (**F**) The top 10 V-J combinations in PBMCs from the 4 patients following decitabine therapy are listed.

The frequencies of the top 10 V-J combinations in these 4 patients before and after decitabine therapy are shown in Figure 3. The detailed information of amino acid and DNA sequences of CDR3 for the highest expansion clonocytes are listed in Table 1. During T cell maturation, the CD4+ and CD8+ T cells are subjected to different selective pressures, and there are biases in CDR3 sequences present in a T cell repertoire, which could distinguish between CD4+ and CD8+ T cells [20, 21]. We observed decitabine-mediated trends for the usage of distinct TCR V and J gene segments, which could partly reflect the change in T-cell differentiation (Figure 4). Generally, the CD4/CD8 ratio tends to be lower in tumor patients than in healthy persons, while we observed an increase in the CD4/CD8 ratio in solid tumor patients following decitabine treatment (Figure 4A). Emerson et al. have reported that TCRβ V7-9 was more characteristic of CD8+ T cells, while TCRβ V5-4, TCRβ V7-2 and TCRβ V18 were more characteristic of CD4+ T cells [20]. In agreement with their results, we observed decreasing TCRβ V7-9 usage frequency and increasing usage frequencies of TCRβ V5-4, TCRβ V7-2 and TCRβ V18 following decitabine therapy in patients UPN1, UPN2 and UPN3 (Figures 4B to 4E). Furthermore, these changes in UPN1 and UPN2 were more significant compared with those in UPN3 and UPN4, indicating a relative higher clonal expansion lineages in HCC patients. We further summarized the top 10 V-J combinations in these patients' blood samples following decitabine therapy, which also suggested that low-dose decitabine could regulate T-cell differentiation. [21]

**Table 1:** Highest expansion clonocytes following decitabine therapy

Patients	AA	Frequency	
		post	pre
UPN1	CATAPGLSYEQYF	0.061386633	0.008880571
UPN2	CSASDHEQYF	0.043817388	0.015202491
UPN3	CASSSLTDLTSYEQYF	0.008112579	0.041222265
UPN4	CASSLSCS~LWGKEYF	0.007189667	2.47E-06

### Low-dose decitabine diversifies the peripheral T cell pool

**Figure 5:**
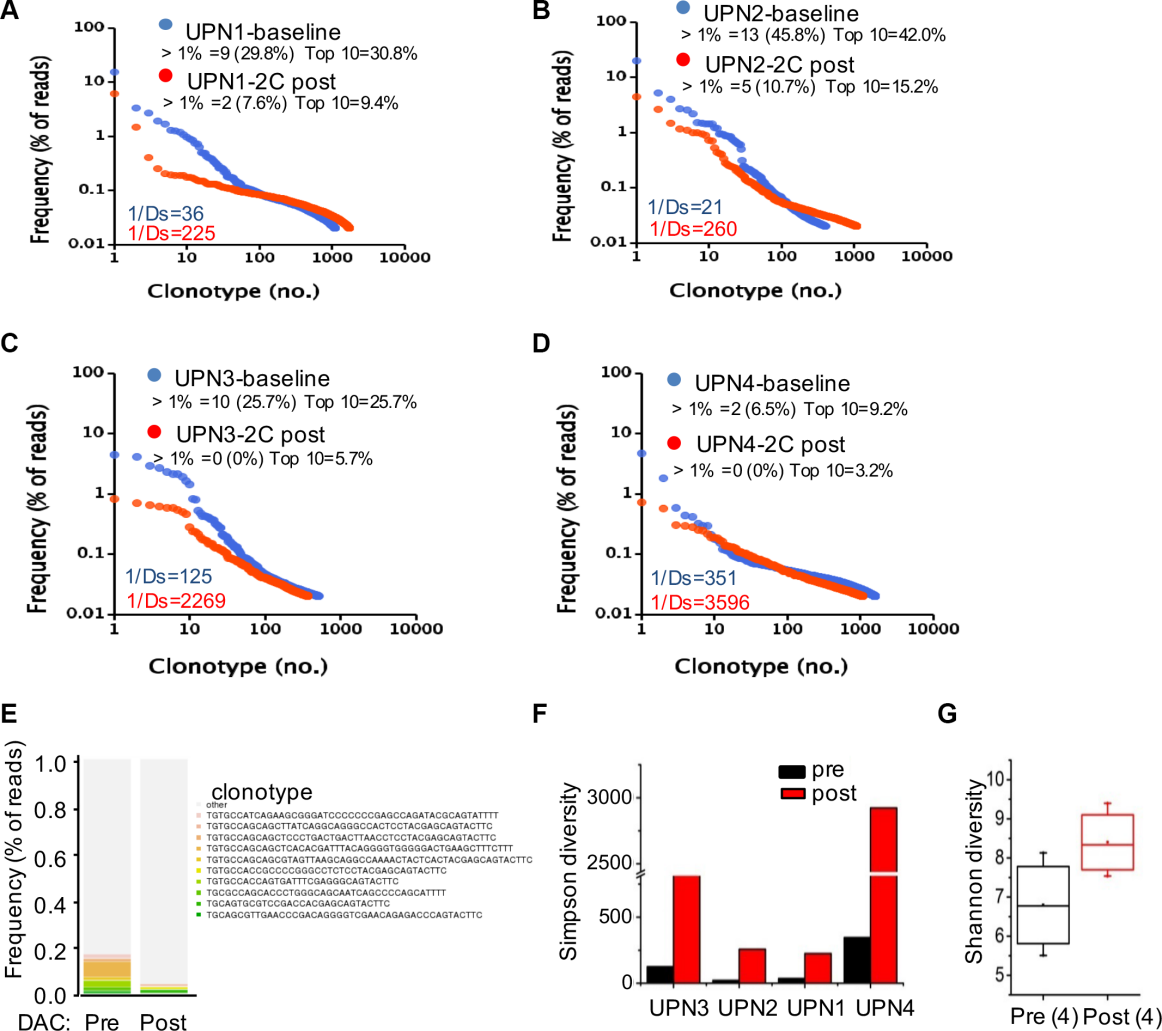
Low-dose decitabine increased TCR diversity in PBMCs (**A**–**D**) Frequency distribution of clonotypes in plots of UPN1 (A), UPN2 (B), UPN3 (C), and UPN4 (D) at baseline (in blue) and after 2 cycles of decitabine therapy (in red). Each dot represents a distinct TCRβ clonotype. Clonotypes are presented at frequencies > 1% and the top 10 clonotypes with their cumulative frequency (percentage of reads). Values in the lower-left corner depict TCR diversity. (**E**) The 10 most frequent clonotypes in the 4 patients at baseline (pre) and after 2 cycles of decitabine therapy (post) are indicated in color and listed at the right; the remaining clonotypes are grouped in black. (**F**) TCR Simpson diversity of the 4 patients after 2 cycles of decitabine therapy compared to that of the baseline. (**G**) The average T cell repertoire Shannon diversity of the 4 patients before and after decitabine therapy.

The frequency of each clonotype was determined within the libraries of all of the TCR reads. In the baseline level in patient UPN1 prior to decitabine therapy, the frequencies of 9 clonotypes were over 1% (altogether 29.8%), the most abundant clonotype contained 15.4% of all reads, and the top 10 clonotypes constituted approximately 30.8% of all reads. In contrast, in the 2 cycles following decitabine therapy, the frequency of only 2 clonotypes was over 1% (altogether 9.4%), the most frequent clonotype included 6.1% of reads, and the top 10 clonotypes constituted 9.4% of reads (Figure 5A). Similar alterations were detected in the other 3 patients (Figure 5B to 5D). The clonotype from all patients after low-dose decitabine treatment was markedly different from that before treatment (Figure 5E).

We used both the Simpson (1/Ds) and Shannon diversity indices to quantify the TCR repertoire diversity. The Simpson index ranges from 1 to ∞, and higher scores on this index represent more richness of the repertoire. As shown in Figure 5F and 5G, an increase in TCR diversity was observed after decitabine treatment compared to the baseline level in all patients. In contrast, ours and other studies have indicated that the TCR repertoire from healthy donors was stable over a long period of time [3, 7, 22], suggesting that the TCR repertoire alterations in patients did not result from temporal dynamics. Comparing the patients' repertoire at the baseline level, we found that UPN1 and UPN2 had a relatively lower TCR diversity than did UPN3 and UPN4, which could reflect the immune status of different tumor patients. Thus, the HCC patients whose TCR repertoires were extremely poor might benefit most from this novel epigenetic treatment strategy.

Unfortunately, due to the limited number of cases, we could not determine the correlation between TCR repertoire diversity and clinical outcome. Anyway, the alteration in TCR CDR3 usage and repertoire diversity observed in PBMCs reflected a new function of the epigenetic agent decitabine in cancer treatment and suggested the prolonged effects of decitabine for the enhancement of the host's intrinsic immune function.

### Genome-wide expression and PRKDC detection in decitabine therapy

**Figure 6:**
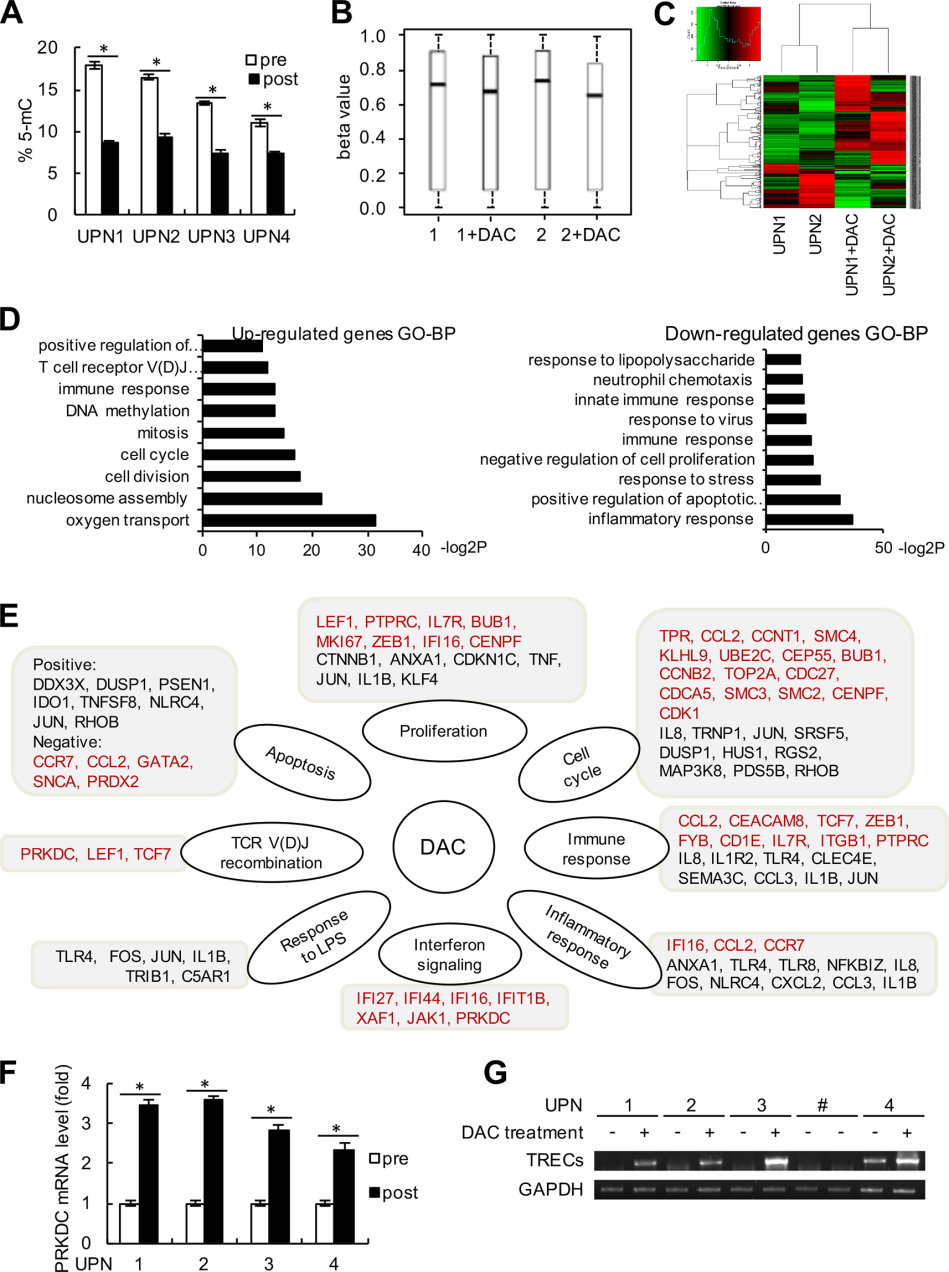
Genome-wide expression alteration and PRKDC induction (**A**) The peripheral T cells were sorted by flow cytometry using anti-CD3 antibody from blood samples in these 4 patients (UPN1 to UPN4). The percentages of 5-mC in T cells from the 4 patients before and after decitabine therapy were detected relative to the total cytosine content. (**B**) Beta value changes in CpG islands in patients UPN1 and UPN2 before and after decitabine therapy. (**C**) Heat map and sample clustering analysis of the differentially expressed genes before and after decitabine therapy in the same patients as (B). (**D**) Significantly enriched up-regulated and down-regulated GO terms in biological process (BP) clusters were analyzed. The data shown are the negative log2 *P*-values within each category. (**E**) Central pathways and differentially expressed genes are shown according to the chip data. Genes in red type indicate the up-regulated genes, and genes in black type indicate the down-regulated genes. (**F**) The relative mRNA expression level of PRKDC was detected by quantitative RT-PCR assay. (**G**) TRECs and GAPDH expression levels were detected in the 4 patients before and after decitabine therapy as described in the “Method” section. UPN# (line 7 and 8) was shown as negative control, who was not received the DAC treatment.

To further investigate the underlying mechanism of decitabine epigenetic therapy in immune reconstitution, we first assessed whether decitabine had direct influence on the immunomodulation of T cells. The peripheral T cells were isolated from the PBMCs of the 4 patients using anti-CD3 antibody by flow cytometry, and the global DNA methylation levels were detected. As shown in Figure 6A, global DNA hypomethylation following decitabine treatment was demonstrated by the downward trend in methylation of LINE-1 repetitive elements. In addition, we analyzed the gene expression profiles in the PBMCs from 2 representative solid tumor patients (UPN1 and UPN2) following 2 cycles of decitabine treatment compared with those before treatment. First, the Infinium Human Methylation450 BeadArray (Accession number GSE80377) indicated that the DNA methylation levels decreased after decitabine treatment, suggesting long-term memory for DNA demethylation (Figure 6B). Through an Affymetrix GeneChip (HG-U133A) analysis (Accession number GSE72686), 430 DEGs were identified, among which 67.7% (291 genes) were up-regulated whereas 32.3% (139 genes) was down-regulated (Figure 6C). As shown in Figure 6D, the up-regulated genes were associated with oxygen transport, cell division, cell cycle, mitosis, nucleosome assembly, DNA methylation and TCR V(D)J recombination, while the enriched biological processes for the down-regulated genes were mainly involved in inflammatory response, positive regulation of apoptosis and negative regulation of cell proliferation (Figure 6D). Decitabine-controlled biological processes with potential regulated genes are listed in Figure 6E.

DNA-dependent protein kinase catalytic subunit (PRKDC), also known as DNA-PKcs, is required for the non-homologous end joining (NHEJ) pathway of DNA repair, and the V(D)J recombination process. PRKDC plays an important role in promoting immune system diversity, and PRKDC knockout mice were reported to show severe combined immunodeficiency since the impaired V(D)J recombination. Notably, the chip array suggested that PRKDC might be up-regulated following decitabine therapy (Figure 6E). We further used Q-PCR assays and validated that the expression of PRKDC was augmented in peripheral T cells from these 4 patients after decitabine treatment compared with those before epigenetic therapy (Figure 6F). In addition, we detected the TCR excision circles (TRECs), which could indirectly assess the thymus output of newly derived T cells [23]. As shown in Figure 6G, an increase in TRECs levels was observed in UPN1 to UPN4 following decitabine treatment. These data indicated that decitabine therapy could modulate TCR rearrangement and increase T cell repertoire diversity.

## DISCUSSION

oneda [24] TCR diversity is one of the most important characteristics of the adaptive immune system, which permits the host to fight a variety of foreign antigens. Although antigen-specific CD8+ cytotoxic T lymphocytes (CTLs) were easily detectable, their pathogen-clearing capacity was lost. Moreover, the dominant T cell clonotype was unable to recognize the variant peptides *in vitro*. Thus, the formation and maintenance of a diverse epitope-specific TCR repertoire would be necessary in tumor therapy [25–28]. Our study demonstrated that low-dose decitabine treatment was able to increase the richness of the peripheral immune repertoire in solid tumor patients. Thus, low-dose decitabine therapy could not only be an adjuvant treatment in sensitizing chemotherapy but also function to directly modulate the organizer's immune system, revealing the promising clinical value of this novel epigenetic therapy.

Recently, it has been reported that CTLA-4 blockade can promote antitumor T cell immunity and induce the diversification of the peripheral T cell repertoire. Notably, the maintenance of high-frequency clonotypes was associated with improved clinical outcomes [9, 22]. In addition, radiation could reconstitute the TCR repertoire of intratumoral T cells, and the combination of radiation and immune checkpoint inhibitors promoted immune response in cancers [29]. Moreover, administration of recombinant human IL-7 in humans caused a marked broadening of circulating TCR diversity with a sustained increase in naïve T cells [30]. In another preclinical trial, rIL-21 treatment improved TCR diversity, boosted T-cell proliferation and re-established effective immunity in the elderly [31]. In our decitabine clinical studies, increased peripheral T cell diversity that correlated with clinical response was observed, suggesting that decitabine-primed T cells could accommodate tumor heterogeneity and respond to novel tumor antigens. To further define the relevance between post-therapy TCR repertoire normalization and tumor remission, an extended sample size was needed, and we predicted that the diversity of T cells in the PR group would be higher than that of SD and PD groups. We have shown that low-dose decitabine therapy enhanced peripheral T cell immune reconstitution and broad circulating TCR diversity. However, due to the difficulty in getting the tumor infiltration lymphocytes, based on our present data, whether the tumor-specific T cell clonotypes with more cytotoxic ability were induced is unclear.

The mechanisms for peripheral TCR repertoire enrichment in response to decitabine treatment could be multiple. Decitabine could directly regulate TCR V(D)J recombination and T cell differentiation. Moreover, since the expansion of TCR variable β subsets was correlated to antigenic stimulation and decitabine treatment could increase the expression of tumor antigens [32]; it is likely that the diverse TCR clonotypes could be generated due to the neoantigens. In addition, decitabine may perhaps lead to the reactivation of some original memory TCR clones. Interestingly, the HCC patients had dramatically less TCR diversity as compared with that of the ovarian cancer donors (Figures 5A and 5B vs 5C and 5D). The HCC cells often have hepatitis B virus (HBV)-DNA integration and can be targeted by HBV-induced specific T cells. The intrahepatic immune suppressive environment might result in the dysfunction or exhaustion of the antigen-specific T cells in HCC patients [33]. The epigenetic modifying agents were recently reported to induce the expression of Th1-type chemokines and promote effector T cell trafficking to the tumor environment [34]. Thus, decitabine-based therapy would enhance anti-tumor immunity [23].

## MATERIALS AND METHODS

### Patients and sample collection

This study was approved by the local ethics committee. 4 patients underwent low-dose decitabine therapy at the Chinese PLA General Hospital, and their characteristics are summarized in Table 2. Generally, patients were given 7 mg/m^2^ decitabine by intravenous push for 30 min over consecutive 5 days of each 28-day treatment cycle. Patient UPN1 was treated for low-dose decitabine monotherapy. Patient UPN2 received argon-helium cryosurgery followed by 4 cycles of low-dose decitabine treatment. Patients UPN3 and UPN4 were given 7 mg/m^2^ decitabine for 5 days followed with reduced-dose paclitaxel and carboplatin-based (TC) chemotherapy administered on day 6. Paclitaxel was used at 135 mg/m^2^, and carboplatin was used at (area under the curve, AUC = 5). Patients were assessed using a CT scan or MRI every 2 cycles. All donors were informed of the use of their blood and signed an informed consent. Peripheral blood was collected into heparin-treated vacutainer tubes. PBMCs were isolated by Ficoll-Paque density-gradient centrifugation.

**Table 2:** Patients characteristics

Patients	Age	Sex	Baseline diagnosis	DAC Treatments	Response to DAC therapy (after 2 cycles)
UPN1	54	M	hepatocellular carcinoma	DAC monotherapy	SD
UPN2	58	M	hepatocellular carcinoma	PRFA combined DAC	SD
UPN3	54	F	carcinoma of uterine tube	DAC+chemotherapy	SD
UPN4	42	F	ovarian cancer	DAC+chemotherapy	PR

### Immune repertoire sequencing analysis

Total RNA was extracted using a TRIzol agent, and cDNA was synthesized using the PrimeScript RT Reagent Kit according to the manufacturer's instructions. The TCR β CDR3 region was defined as segment starting with the second conserved cysteine by TCR Vβ 3′ part and ending with the conserved phenylalanine by the TCR Jβ 5′ part, which was amplified and sequenced by BGI Tech (Shenzhen, China) using the previously described protocols [35]. For CDR3 spectratyping, each TCR Vβ fragment was amplified with one of the 23 Vβ-specific primers and a 5′ FAM-labed Cβ primer [36], excluding TCR Vβ10, Vβ19 and Vβ24. The PCR amplification and size distribution of each fluorescent PCR product was performed as follows [36]. The Multiplexed PCR was carried out to amplify all of the possible rearranged TCRβ segments, by using 48 forward primers and 13 reverse primers as previously described [37]. The FASTA format sequence data were analyzed through IMGT (ImMunoGeneTics) database.

### Quantitative real-time PCR (Q-PCR)

Total RNA was isolated using a TRIzol agent, and the cDNA was synthesized by a reverse transcription kit (Invitrogen). For PRKDC gene expression analysis, the relative mRNA levels were normalized to the internal control GAPDH with the 2^−ΔΔCt^ cycle threshold method, by using SYBR Green according to the manufacturer's protocol. The following primers were used in this study: PRKDC forward primer: 5′- TTTCCAGAGATTTCGGTTTGC-3′, PRKDC reverse primer: 5′- AATTTCAACAGAGTAAGGTGCGAT-3′; GAPDH forward primer: 5′- GGGAAGGTGAAGGTCGG AGT-3′, GAPDH reverse primer: 5′- TTGAGGTCAATGA AGGGGTCA-3′.

### TRECs detection by semi-nested PCR

For TRECs analysis, a semi-nested PCR was performed as previously reported [38]. For the first PCR, 200 ng of genomic DNA from PBMC was amplified in a 10 μl reaction system containing external forward and reverse primers. Then a nested PCR was performed with 2 μl of the first PCR products and the internal forward and the same reverse primer. Amplification of TRECs and reference gene GAPDH was conducted in triplicate. The following primers were used in this study: TRECs external forward primer: 5′-gtcatagcttaaaaccctccgagtgacgcacagcc-3′, TRECs reverse primer: 5′-agaccagccccttcgccaaacagccttac-3′, and TRECs internal forward primer: 5′-cgtagtgcctaccaac ctgctacacacttatttcc-3′. GAPDH forward primer: 5′-AATG GGCAGCCGTTAGGAAA-3′, and GAPDH reverse primer: 5′-GCGCCCAATACGACCAAATC-3′.

### Immunofluorescence analysis

The following antibodies were used to detect surface marker expression: CD4-FITC, CD8-PE, CD3-PerCP. All of these antibodies and isotype-matched antibodies were obtained from BD Biosciences. Data acquisition was performed using a FACSCalibur flow cytometer (BD Biosciences). Experiments were conducted in triplicate.

### Statistical analysis

Because of the small sample size in this study, analysis of differences among data groups was performed with the Mann-Whitney test. A *P* value of < 0.05 was considered statistically significant.

## SUPPLEMENTARY MATERIALS TABLES


