# Supplementary material for: DNA demethylating agent decitabine broadens the peripheral T cell receptor repertoire
Source: Oncotarget. 2016 May 13;7(25):37882–92. doi: 10.18632/oncotarget.9352 (PMC5122357; doi:10.18632/oncotarget.9352)
Supplement: Supplementary file 1 [file oncotarget-07-37882-s001.pdf]

DNA demethylating agent decitabine broadens the peripheral T cell receptor repertoire

Supplementary Materials

Supplementary Table S1: Basic sequencing data

Sample	Total input sequences	Bases processed	Total good sequences	Sequencing information utilization (%)	Clonotype number	Out of frame clones (%)	Clone with stops
UPN1-pre	23399855	2810445058	10166914	43.45	20719	29.3	6.04
UPN1-post	21601643	2606968972	8024760	37.15	22913	30.18	6.49
UPN2-pre	23029114	3109435720	18336797	79.62	32956	28.68	6.36
UPN2-post	27225099	3564482749	19718268	72.43	51635	32.28	5.71
UPN3-pre	20435520	2653239398	16008873	78.34	48707	33.48	6.55
UPN3-post	21503583	2741890167	18962896	88.18	120787	38.59	6.05
UPN4-pre	21170000	2550299906	10132095	47.86	29592	39.17	7.95
UPN4-post	23055366	2834896056	13131623	56.96	41455	40.19	6.38

Supplementary Table S2: The frequency of various V, D, J genes of TCRβ in each patient

Sample	UPN1-pre	UPN1-post	UPN2-pre	UPN2-post	UPN3-pre	UPN3-post	UPN4-pre	UPN4-post
TRBD1	0.437593	0.449369	0.585629	0.481434	0.497668	0.482417	0.431062	0.479294
TRBD2	0.562407	0.550631	0.414371	0.518566	0.502332	0.517583	0.568938	0.520706
TRBJ1-3	0.010604	0.012244	0.006165	0.016612	0.007455	0.016398	0.012689	0.0245
TRBJ2-5	0.105545	0.091431	0.051204	0.106191	0.142077	0.136918	0.132343	0.13153
TRBJ1-5	0.030198	0.038798	0.093389	0.031701	0.025499	0.039351	0.031458	0.043887
TRBJ2-2	0.04104	0.050944	0.020729	0.036614	0.049974	0.031379	0.044532	0.045967
TRBJ1-6	0.025982	0.053725	0.022896	0.037356	0.0318	0.054172	0.036205	0.06284
TRBJ1-2	0.049437	0.050586	0.044063	0.063952	0.063419	0.104009	0.045142	0.074317
TRBJ2-6	0.004755	0.012586	0.011264	0.005495	0.004862	0.007114	0.009327	0.00937
TRBJ2-3	0.069103	0.094353	0.132156	0.076033	0.074774	0.068227	0.123263	0.087062
TRBJ1-1	0.084805	0.06412	0.234392	0.053196	0.064815	0.06506	0.100981	0.086004
TRBJ2-1	0.15033	0.192195	0.116689	0.206131	0.196563	0.16636	0.190458	0.163768
TRBJ2-4	0.002833	0.003652	0.002339	0.004673	0.004719	0.005702	0.004088	0.004024
TRBJ1-4	0.042552	0.028767	0.024518	0.01217	0.009944	0.009504	0.019036	0.023135
TRBJ2-7	0.382815	0.3066	0.240196	0.349876	0.324099	0.295805	0.250478	0.243596
TRBV11-1	0.011168	0.002963	0.00069	0.001623	0.000605	0.000673	0.002164	0.001153
TRBV3-1	0.000639	0.00141	0.000811	0.002134	0.000948	0.001427	0.001074	0.001596
TRBV19	0.055228	0.109126	0.054513	0.048675	0.05378	0.051152	0.047199	0.049546
TRBV9	0.060286	0.033867	0.017866	0.024175	0.064213	0.030242	0.026507	0.036907
TRBV6-2	0.006941	0.00529	0.003255	0.008312	0.003878	0.002073	0.003183	0.001499
TRBV30	0.000146	0.000532	0.000511	0.000573	0.00008	0.000216	0.000199	0.000487
TRBV6-5	0.007108	0.016284	0.008597	0.017691	0.025795	0.007086	0.012945	0.013449
TRBV7-3	0.044469	0.029009	0.018314	0.054694	0.018194	0.023404	0.088986	0.043482
TRBV7-4	0.001437	0.001912	0.000704	0.000967	0.001649	0.001483	0.00479	0.001399
TRBV6-6	0.004712	0.007115	0.00448	0.004031	0.002256	0.002256	0.005744	0.00587
TRBV25-1	0.023078	0.027657	0.008594	0.028789	0.036708	0.046858	0.045471	0.028155
TRBV18	0.000048	0.000111	0.000138	0.000808	0.00009	0.000232	0.000233	0.000187
TRBV6-3	0.006941	0.00529	0.003255	0.008312	0.003878	0.002073	0.003183	0.001499
TRBV5-1	0.034715	0.043221	0.025294	0.033003	0.034972	0.018227	0.034074	0.038247

TRBV12-4	0.027961	0.022087	0.015358	0.019476	0.016581	0.012363	0.01159	0.022289
TRBV4-1	0.003298	0.004914	0.013395	0.006607	0.002967	0.00239	0.002669	0.005911
TRBV7-1	0.00005	0.000024	0.000034	0.000001	0.000003	0.000024	0.000018	0.000075
TRBV15	0.017384	0.028289	0.011738	0.044012	0.043555	0.065403	0.035757	0.033639
TRBV11-3	0.00151	0.006203	0.002578	0.005662	0.004124	0.004222	0.003119	0.003047
TRBV6-1	0.00505	0.012586	0.003837	0.007889	0.005648	0.004138	0.011233	0.01237
TRBV10-1	0.016826	0.015604	0.039016	0.013846	0.014495	0.022692	0.022652	0.019335
TRBV29-1	0.04425	0.051447	0.017048	0.042645	0.086974	0.092434	0.060436	0.055566
TRBV7-7	0.005565	0.007707	0.004297	0.006935	0.006837	0.007463	0.011033	0.007741
TRBV4-3	0.00506	0.003561	0.001063	0.003968	0.001466	0.001369	0.00243	0.000125
TRBV23-1	0.000005	0.000016	0.000005	0.000004	0.000025	0.000006	0.000007	0.00009
TRBV20-1	0.107693	0.148652	0.102508	0.190904	0.109942	0.123554	0.150775	0.169168
TRBV28	0.001414	0.004671	0.003191	0.003343	0.00153	0.001687	0.002415	0.002952
TRBV6-7	0.000014	0.000562	0.000127	0.000415	0.00015	0.000093	0.000449	0.000437
TRBV6-4	0.002775	0.005493	0.005465	0.010999	0.001206	0.001445	0.003739	0.010882
TRBV2	0.038481	0.049264	0.019515	0.036836	0.034666	0.079777	0.054269	0.074585
TRBV27	0.000714	0.002501	0.00196	0.001986	0.000434	0.00129	0.000881	0.001282
TRBV7-2	0.042734	0.059995	0.017997	0.041522	0.060274	0.064234	0.082211	0.04663
TRBV5-8	0.001015	0.002503	0.003268	0.002914	0.002114	0.001489	0.002332	0.003596
TRBV13	0.001171	0.003608	0.001683	0.004026	0.001658	0.00236	0.002225	0.002195
TRBV21-1	0.000165	0.000283	0.000006	0.00013	0.000008	0.000028	0.000068	0.000088
TRBV12-5	0.000493	0.001236	0.0004	0.0017	0.00034	0.000456	0.001137	0.001497
TRBV5-5	0.002882	0.004431	0.007678	0.004102	0.002281	0.003133	0.003953	0.005432
TRBV7-9	0.063351	0.05482	0.27284	0.061104	0.094739	0.068624	0.053614	0.066034
TRBV10-3	0.038307	0.03269	0.095071	0.04414	0.045518	0.079688	0.047773	0.061161
TRBV5-6	0.012504	0.01641	0.014403	0.011829	0.011511	0.006764	0.015907	0.008859
TRBV7-6	0.013188	0.016712	0.007264	0.018073	0.016855	0.01767	0.019151	0.017641
TRBV10-2	0.02987	0.017549	0.068264	0.013316	0.01666	0.019042	0.013666	0.013357
TRBV16	0.000912	0.000326	0.000143	0.000444	0.000226	0.000319	0.000333	0.000322
TRBV5-4	0.008151	0.016067	0.005178	0.009483	0.002969	0.004594	0.009106	0.014595
TRBV7-8	0.024175	0.028645	0.010389	0.02045	0.074783	0.03699	0.024852	0.026275
TRBV24-1	0.172016	0.05063	0.051696	0.061547	0.037904	0.049155	0.044048	0.047456
TRBV12-3	0.027961	0.022087	0.015358	0.019476	0.016581	0.012363	0.01159	0.022289
TRBV11-2	0.002964	0.009375	0.00763	0.007987	0.007312	0.008593	0.008271	0.009544
TRBV4-2	0.000861	0.002379	0.004236	0.003972	0.005938	0.002492	0.002397	0.003041
TRBV14	0.022313	0.012884	0.02834	0.044471	0.024679	0.014254	0.008141	0.007017
